# Identification of clinically-useful cut scores of the Traumatic Injuries Distress Scale (TIDS) for predicting rate of recovery following musculoskeletal trauma

**DOI:** 10.1371/journal.pone.0248745

**Published:** 2021-03-23

**Authors:** David M. Walton, James M. Elliott, Joshua Lee, Mohamad Fakhereddin, Wonjin Seo

**Affiliations:** 1 School of Physical Therapy, Faculty of Health Sciences, Western University, London, Canada; 2 Northern Sydney Local Health District & Faculty of Medicine and Health, The University of Sydney, Sydney, Australia; University Hospital Zurich, SWITZERLAND

## Abstract

**Objective:**

The Traumatic Injuries Distress Scale (TIDS) is a 12-item self-report tool intended for prognostic risk phenotyping in people with acute musculoskeletal (MSK) trauma. The initial validation study showed good associations with outcomes 12 weeks later in a cohort of 72 acutely injured patients from one region in Canada. This study aims to provide further clinical utility through identification of meaningful cut scores in a larger, mixed geography sample, and expands the prediction window from 12 to 52 weeks.

**Methods:**

Data were drawn from databanks in London, Canada and Chicago, United States. Participants were recruited within 3 weeks of non-catastrophic MSK trauma and followed for 12 months. Using outcomes trajectories, the TIDS underwent linear regression-based analysis to predict 52-week outcomes, and area under the receiver operating characteristic curves to identify discriminative accuracy and meaningful cut scores.

**Results:**

N = 224 participants with acute trauma were followed and both %Interference and Pain Severity were captured at intake and 3 follow-ups to establish curvilinear recovery trajectories. The TIDS explained significant variance in both the interference and severity outcomes after controlling for sex, region of injury, and baseline scores. ROC analysis revealed significant discriminative accuracy for predicting both the trajectories and the distal outcomes over 52 weeks. The TIDS was more accurate for identifying the low-risk than high-risk patients.

**Conclusion:**

The TIDS is a useful tool for ‘ruling out’ high risk of poor outcome in a mixed sample of participants from two different countries.

**Impact statement:**

The TIDS will be a useful tool for clinicians to predict the rate of recovery by displaying meaningful cut-scores for their patients after an acute musculoskeletal injury. This could lead to reduced burden of care for low risk patients and more informed treatment options for higher risk patients.

## Introduction

Prospective research indicates that 10 to 50% of people who experience an acute non-catastrophic musculoskeletal (MSK) injury will report persistent pain-related symptoms or interference 6 to 24 months following the event. This includes whiplash associated disorder (WAD) [1, 2], low back injuries [3, 4], and distal radius fractures [5], among others. Evidence consistently demonstrates 3 recovery trajectories following non-catastrophic trauma: i) fully recovered / no persistent symptoms, ii) moderate or delayed recovery / some persistent symptoms, or iii) little-to-no recovery / significant persistent symptoms [2, 6]. These findings are becoming adequately consistent across body regions and outcomes that a 3-class approach to prognosis, rather than traditional dichotomous conceptualizations of low vs high risk, seems empirically justified.

Owing to the personal and social burden of chronic pain, there have been several attempts to create screening tools or protocols for the ‘high risk’ patient with the intention of allowing early identification and intervention to prevent chronicity. These include protocols for WAD [7], low back pain [8], and generalized MSK trauma [9, 10]. The available published tools have shown predictive accuracy in discriminating between low and high-risk patients, from 75–85% accurate dependent on the sample and outcome predicted [11–13]. The majority of available tools have been designed for use in a specific patient population or those with injuries in specific regions. Non region-dependent tools are fewer, though offer the potential benefit of requiring familiarity with only one screening tool applicable to all body regions. One such attempt was that of Lentz and colleagues [9] who created a yellow-flags screening tool for use in multi-region MSK injury. The tool showed sound concurrent associations with other metrics of psychological distress, and in a follow-up longitudinal study was able to explain significant linear variance in 12-month recovery outcomes in patients with both acute and chronic symptoms [11].

Another non region-dependent tool is the Traumatic Injuries Distress Scale (TIDS), a 12-item self-report tool intended to capture the likelihood of, and reasons for, non-recovery in all-cause MSK trauma. The TIDS was designed to be relevant in the acute post-trauma period and can be interpreted as both a total summed score or as 3 separate subscales: *Uncontrolled Pain*, *Negative Affect*, *and Intrusion/Hyperarousal*. In the initial development study, the TIDS demonstrated sound factorial validity and in samples of 45 to 72 acutely-injured (<3 weeks from injury) participants, showed significant linear associations with 5 different outcomes 12 weeks later [14].

While the initial analyses were promising, the sample was limited to a mid-sized Canadian city, correlation and regression analyses are difficult to translate clinically, and predicting outcomes beyond 12 weeks post-injury will add further utility to such a tool. Therefore the purpose of this study was to explore the 12-month predictive validity of the TIDS in a large mixed sample of acutely-injured patients recruited from two different countries (Canada and the United States) and to identify meaningful cut-scores and associated accuracy for prediction of the recovery trajectory and distal outcomes of acutely injured patients. A secondary purpose was to explore the prognostic accuracy of the TIDS for differential functioning when disaggregated by sex, age, body mass index, or geographical region.

## Methods

Data for this analysis were drawn from two different longitudinal acute trauma cohorts: the Systematic Merging of Biology, Mental Health, and Environment (SYMBIOME) longitudinal cohort study (clinicaltrials.gov ID No. NCT02711085), and the Neuromuscular Mechanisms Underlying Poor Recovery from Whiplash Injuries (NMUPRWI) study (ClinicalTrials.gov Identifier: NCT02157038). The methods for data collection have been described previously [15]. Briefly, after being medically cleared and discharged from emergency or acute trauma care units for care of symptoms after a distinct MSK injury, potentially eligible participants (<4 weeks from injury, not requiring surgery or hospitalization, at least 18 years old and able to speak conversational English) were screened and consented by a research assistant. Initial participant data were collected through a robust package of self-report forms completed within 24 hours from inception. Amongst those were study specific patient data and demographics (age, sex-at-birth, education, employment, income, height and weight), the TIDS, and either the Brief Pain Inventory (BPI, Ontario cohort) or the Neck Disability Index (NDI, Illinois cohort). The TIDS has been described above and in a prior publication [14]. The BPI is a widely used patient-reported outcome that includes two subscales: Pain Severity (reported as a mean /10) and Pain Interference (/70). It has been used and adequately validated across several pain conditions including MSK pain [16]. The NDI is the most widely used neck-specific disability questionnaire globally [17]. It has adequate evidence as a valid and reliable tool for capturing pain-related interference in people with neck pain specifically [18]. Both the BPI and NDI can be reported as a percentage by dividing summed score by maximum possible score. The Chicago cohort was comprised exclusively of people with traumatic neck pain and used the NDI as the primary outcome, while the London cohort was comprised of mixed-region injuries and used the BPI as the primary outcome.

Data were captured at inception, and again at 2–4 weeks, 12 weeks, and 52 weeks (12 months) post-injury.

The NDI and BPI Interference scores were combined as indicators of ‘pain-related interference’ and percent scores (%Interference) were used to identify growth trajectories through maximum likelihood estimation (MLE)-based growth mixture modeling (GMM) reported in a prior paper [15]. Through that analysis 3 recovery trajectories were identified as defined by interference outcomes over 12 months: *Rapid Recovery*, *Delayed Recovery*, and *Little or No Recovery*. By 12 months both the Rapid and Delayed Recovery groups reported mean interference scores <1%, while those in the Little or No Recovery group reported mean 18% persistent interference. A similar GMM approach for the Pain Severity scores revealed two trajectories: *Rapid Recovery* (mean NPRS <2/10 by 3 months) and *Little or No Recovery* (mean NPRS = 5.1/10 at 12 months). Through these prior analyses all current participants were classed into one of three %Interference trajectories, one of two Pain Severity trajectories, and by %Interference scores at just the 12-month follow-up (<5% = *No Interference* (full recovery), 5–20% = *Moderate Interference*, and >20% = *Significant Interference*).

### Missing data

Where <20% of scores on a baseline questionnaire were missing, those responses were replaced with the mean. Where 20% or more were missing, those questionnaires were excluded from the analysis. Where 12-month Interference or Severity data were missing, final values were estimated from the MLE-LGCA using lines of best-fit, as described in our prior paper [15]. Only participants with at least 2 of 3 follow-up data points were included in these analyses.

### Analysis

#### Pre-analysis: Establishing equivalency of disability outcomes

As the cohorts used two different outcomes for tapping the construct of ‘pain-related functional interference’, we conducted two pre-analyses and one post-analysis to explore construct equivalence and justify pooling into a single ‘%Interference’ metric. In the pre-analysis we first compared %BPI in the SYMBIOME cohort to %NDI in the NMUPRWI cohort using an independent samples t-test, where no significant difference was considered acceptable. Second we used the consistent pain severity item across the two cohorts and conducted a bootstrapped bivariate correlational analysis to estimate Pearson’s r coefficient with 95% confidence intervals (95%CI), where confidence intervals from one analysis that overlapped the point estimate (r) of the second cohort was considered acceptable for construct equivalence. In the post-analysis, the prognostic accuracy established in the cohort overall was also conducted on the two cohorts separately, where again non-significant differences in predictive accuracy (confidence intervals of area under the receiver operating characteristic curve) was considered evidence of adequate similarity.

#### Primary analyses–predicting outcomes

Demographic data of the full dataset were explored descriptively (means or proportions). Assuming acceptable evidence of construct similarity from the pre-analysis, two stepwise linear regression equations were created, one for 12-month %Interference (0–100) and one for 12-month Pain Severity (0–10). Independent variables were (in order of entry): sex, age, body mass index (BMI kg/m^2^), region of injury (axial spine vs. extremity), baseline %Interference or Severity score (per the dependent variable being predicted), and baseline TIDS total score. Significance in ΔF for retention was p<0.05. Assuming homoscedasticity and good model fit, the unique variance in both key outcomes explained by the TIDS was evaluated through the significance of ΔF and Δr^2^ after controlling for the prior variables.

Assuming linear predictive validity from regression, each participant was then assigned to one of the previously derived recovery trajectory classes or 12-month outcome classes. After affirming acceptable normality in data distribution, mean TIDS scores were compared for differences across the primary outcome categories with independent samples t-test (Pain Severity trajectories) or one-way Analysis of Variance (ANOVA) with Tukey’s post-hoc test to further explore any significant effects.

#### Identification of cut scores

Receiver operating characteristics (ROC) curves were then constructed and the area under the curves (AUC) were calculated as omnibus indicators of discriminative accuracy for each outcome, where an AUC = 0.50 indicated discrimination no better than chance. For the 3-category %Interference outcomes, the index category was ‘non-recovery’ (minimal or no recovery trajectory, or significant interference outcome) compared to the other two ‘recovery’ classes. The AUC analysis was also conducted with the sample stratified by sex-at-birth (male or female), median sample age (38 years), median sample BMI (25.1 kg/m^2^), and geographical region (Ontario vs. Illinois) to identify any differential functioning across groups. AUC was compared across person variables for significant differences in discriminative accuracy by using an independent samples χ^2^ test in MedCalc v19.2.0 software (Ostend, Belgium).

Finally, meaningful cut scores for the TIDS Total score were derived using the coordinates of those ROC curves. Specificity was prioritized for the ‘Low Risk’ cut score (those scoring under that threshold are very likely not going to develop persistent problems) as a beta error would mean withholding treatment from someone who might otherwise benefit. That priority was loosened slightly for the ‘High Risk’ threshold, as erroneously deeming someone as high risk would mean they are offered treatment when none is needed, an error that for rehabilitation interventions is less problematic. We further opted for thresholds that captured at least 1/5^th^ (20%) of the sample to optimize utility of the scale as a clinical screening tool.

Sample size for this analysis was not determined *a priori*, rather all available data were used.

## Results

The combined primary database included 224 participants with acute MSK trauma. The sample was 66.4% female, mean of 39.3 years old (range 18 to 66 years), and BMI of 26.0 kg/m^2^ (range 14.4 to 51.5 kg/m^2^). Table 1 presents participant demographics and baseline values for TIDS and primary outcomes. In the pre-analysis step exploring construct equivalence across tools, there was no significant difference in %Interference between the Ontario (mean = 38.4%, SD = 23.9%) and Illinois (mean = 36.3%, SD = 16.7%) cohorts (p = 0.44). Bootstrapped correlation coefficients were not significantly different between severity with %BPI (r = 0.67, 95%CI = 0.54 to 0.76) in the Ontario cohort, and severity with %NDI (r = 0.55, 95%CI = 0.40, 0.69) in the Illinois cohort. Accordingly, we were comfortable pooling the two metrics into a shared ‘%Interference’ outcome.

**Table 1:** Baseline characteristics and study data.

N = 224	Proportion or Mean (Range)
**Sex (% female)**	66.4%
**Age (mean, range)**	39.3 years (18 to 66)
**BMI (mean, range)**	26.0 (14.4 to 51.5) kg/m^2^
**Region of injury**	
** Axial spine (neck, back)**	60.3%
** Upper/Lower extremity**	39.7%
**TIDS (mean, range)**	
** Total (/24)**	8.1 (0 to 24)
** Uncontrolled Pain (/8)**	3.3 (0 to 8)
** Negative Affect (/12)**	3.7 (0 to 12)
** Intrusion/Hyperarousal (/4)**	1.1 (0 to 4)
**Pain Severity /10 (mean, range)**	4.7 (0 to 10)
**Pain-related Functional Interference /100% (mean, range)**	37.6% (0 to 96%)

BMI = Body Mass Index, TIDS = Traumatic Injuries Distress Scale.

### Primary analyses

Table 2 shows the results of stepwise linear regression for predicting each outcome. Neither age nor BMI showed significant association in any model. Sex, region of injury, baseline %Interference score and baseline TIDS Total were retained in the 12-month %Interference prediction model, collectively explaining 29.9% of variance. For Pain Severity prediction, only region of injury and acute TIDS total score were significant predictors, explaining 22.3% of variance in 12-month score.

**Table 2:** Results of stepwise linear regression for 52-week % Interference and 52-week Pain Severity.

	Β (std. *β*)	ΔR^2^ (% variance)	ΔF	p
**52-week %Interference**				
**Sex**	2.32 (0.09)	2.7%	5.42	0.02
**Region**	-9.71 (-0.38)	18.8%	46.70	<0.01
**Acute %Interference**	0.07 (0.13)	6.3%	19.97	<0.01
**TIDS**	0.44 (0.21)	2.1%	5.75	0.02
**Total**		29.9%		
**52-week Pain Severity**				
**Sex**	0.40 (0.08)	2.8%	3.26	0.07
**Region**	-1.11 (-0.25)	12.6%	16.86	<0.01
***Acute Pain Severity***	*0*.*05 (0*.*05)*	*2*.*0%*	*2*.*72*	*0*.*10*
**TIDS**	0.11 (0.27)	4.9%	7.00	0.01
**Total**		22.3%		

Only significant predictors are retained. Sex is coded as 1 = male, 2 = female. Region is coded as 1 = axial, 2 = extremity. TIDS is reported as total score /24. *Italics*: Acute Pain Severity did not contribute significant unique variance to 52-week outcome prediction. It is included in the table for comparison purposes only.

Having established linear predictive associations, participants were then categorized as: Rapid Recovery (n = 74), Delayed Recovery (n = 59), and No or Minimal Recovery (n = 91) based on the previously derived %Interference classes. For the pain trajectories, n = 172 were classed in the Rapid Recovery class, and n = 36 were in the Minimal or No Recovery class. For distal 12-month outcomes, 114 (48.6%) were considered ‘recovered’ (<5% interference), 73 (34.5%) were classed as ‘mild-to-moderate interference’ (5–20% interference), and 35 (16.8%) were classed as ‘significant persistent interference’ (>20% interference). Table 3 provides the means and 95% confidence intervals for the TIDS total and subscale scores across the levels of the 3 primary outcomes. T-test or ANOVA revealed significant main effects across the different recovery classes for each of the TIDS Total and subscale scores (all F significant at p<0.05 level). Through Tukey’s post-hoc test all 4 TIDS metrics showed significantly lower values in the lowest risk (rapid recovery or no disability) recovery groups compared to the others. The *Total* score and *Intrusion/Hyperarousal* subscale were significantly different across all 3 levels of %Interference trajectories, and *Total* score was significantly different across the 3 levels of 12-month %Interference point outcome.

**Table 3:** Group means and 95% confidence intervals for TIDS total score and 3 subscales compared across levels of recovery trajectory (<3 weeks to 12 months) or cross-sectional 12-month distal outcome.

	Total	Uncontrolled Pain	Negative Affect	Intrusion/ Hyperarousal
**Interference Trajectories**				
**Rapid Recovery (n = 74)**	4.1 (3.3, 4.9)^1^	1.9 (1.5, 2.3)^2^	1.9 (1.5, 2.4)^2^	0.3 (0.1, 0.4)^1^
**Delayed Recovery (n = 59)**	8.6 (7.3, 9.9)	3.5 (2.8, 4.1)	4.0 (3.3, 4.8)	1.1 (0.8, 1.4)
**No or Minimal Recovery (n = 91)**	11.0 (9.8, 12.2)	4.2 (3.7, 4.7)	4.8 (4.2, 5.5)	1.8 (1.5, 2.1)
**Pain Trajectories**				
**Rapid Recovery (n = 172)**	7.3 (6.5, 8.1)^3^	3.0 (2.7, 3.5)^3^	3.3 (2.9, 3.7)^3^	1.0 (0.8, 1.2)^3^
**No or Minimal Recovery (n = 36)**	12.9 (11.0, 14.8)	4.8 (4.1, 5.6)	5.6 (4.4, 6.8)	1.9 (1.5, 2.4)
**Distal (52-week) scores**				
**<5% disability (n = 114)**	6.1 (5.2, 7.0)^1^	2.7 (2.3, 3.1)	2.8 (2.3, 3.2)^2^	0.6 (0.4, 0.8)^2^
**5–20% Disability (n = 73)**	9.0 (7.6, 10.4)	3.1 (2.6, 3.7)	4.1 (3.3, 4.9)	1.6 (1.3, 1.9)
**>20% Disability (n = 35)**	12.3 (10.5, 14.2)	5.2 (4.4, 5.9)^4^	5.4 (4.3, 6.4)	1.7 (1.2, 2.3)

1: Significant main effect of ANOVA across 3 levels of ‘class’, Tukey’s post-hoc reveals that class means are significantly different between all 3 classes.

2: Significant main effect of ANOVA across the 3 levels of ‘class’. Tukey’s post-hoc reveals significant differences between the ‘Rapid Recovery’ trajectory or ‘No Disability’ 12-month class, and the other two groups. No significant difference between the other groups.

3: Mean differences between the two pain trajectory groups are significant (p<0.01 level).

4: Significant main effect of ANOVA across the 3 levels of ‘class’, Tukey’s post-hoc reveals that baseline mean TIDS score in the ‘12-month Significant Persistent Disability’ class is higher than the other two classes.

### Identification of cut scores

Table 4 presents the AUC when discriminating between the primary outcomes. Similar to the ANOVA-based analyses, TIDS Total score showed significant discriminative accuracy (AUC p < 0.05) between all classes, while each of the subscales showed significant discriminative accuracy between at least two trajectory/outcomes groups. When the sample was disaggregatd by sex, age, or BMI, AUC analysis when showed no significant difference in prognostic accuracy between the groups (χ^2^ for AUC all p>0.10). Diagnostic accuracy of the TIDS total score was also consistent across the two cohorts (Ontario %BPI as outcome: AUC = 0.71, 95%CI = 0.59 to 0.83; Illinois %NDI as outcome: AUC = 0.65, 95%CI 0.53 to 0.77).

**Table 4:** Area under the receiver operating characteristic curve (AUC) for all relevant comparisons as indicators of discriminative accuracy.

Index	Comparator	Total	Uncontrolled Pain	Negative Affect	Intrusion/ Hyperarousal	
*Interference Trajectories*						
Minimal or No Recovery (91)	Rapid or Delayed Recovery (133)^1^	**0.75 (0.69, 0.82)**	**0.70 (0.63, 0.77)**	**0.69 (0.62, 0.76)**	**0.73 (0.66, 0.80)**	
Minimal or No Recovery (91)	Rapid Recovery (74)	**0.85 (0.79, 0.91)**	**0.79 (0.72, 0.86)**	**0.78 (0.71, 0.85)**	**0.80 (0.73, 0.87)**	
Minimal or No Recovery (91)	Delayed Recovery (59)	**0.63 (0.54, 0.72)**	0.59 (0.50, 0.69)	0.58 (0.49, 0.67)	**0.64 (0.54, 0.73)**	
*Pain Trajectories*						
Minimal or No Recovery (36)	Rapid Recovery (172)	**0.77 (0.69, 0.85)**	**0.75 (0.67, 0.82)**	**0.72 (0.63, 0.81)**	**0.70 (0.60, 0.79)**	
*Interference 52-week Outcomes*						
Significant Disability (35)	No or Mod. Disability (185)^1^	**0.76 (0.68, 0.84)**	**0.77 (0.69, 0.85)**	**0.71 (0.62, 0.80)**	**0.64 (0.53, 0.74)**	
Significant Disability (35)	No Disability (114)	**0.81 (0.73, 0.90)**	**0.78 (0.70, 0.87)**	**0.76 (0.67, 0.85)**	**0.71 (0.60, 0.81)**	
Significant Disability (35)	Mod. Disability (73)	**0.67 (0.56, 0.77)**	**0.74 (0.64, 0.84)**	**0.63 (0.52, 0.74)**	0.52 (0.41, 0.64)	

1: For the %Interference Trajectories and Distal Outcomes, the first row compares the Little or No Recovery group against the other two groups combined.

Numbers in parentheses are sample size for each comparison. Bolded values indicate discriminative accuracy significantly greater than chance (AUC = 0.50). BPI = Brief Pain Inventory, Interference subscale.

**Fig 1:**
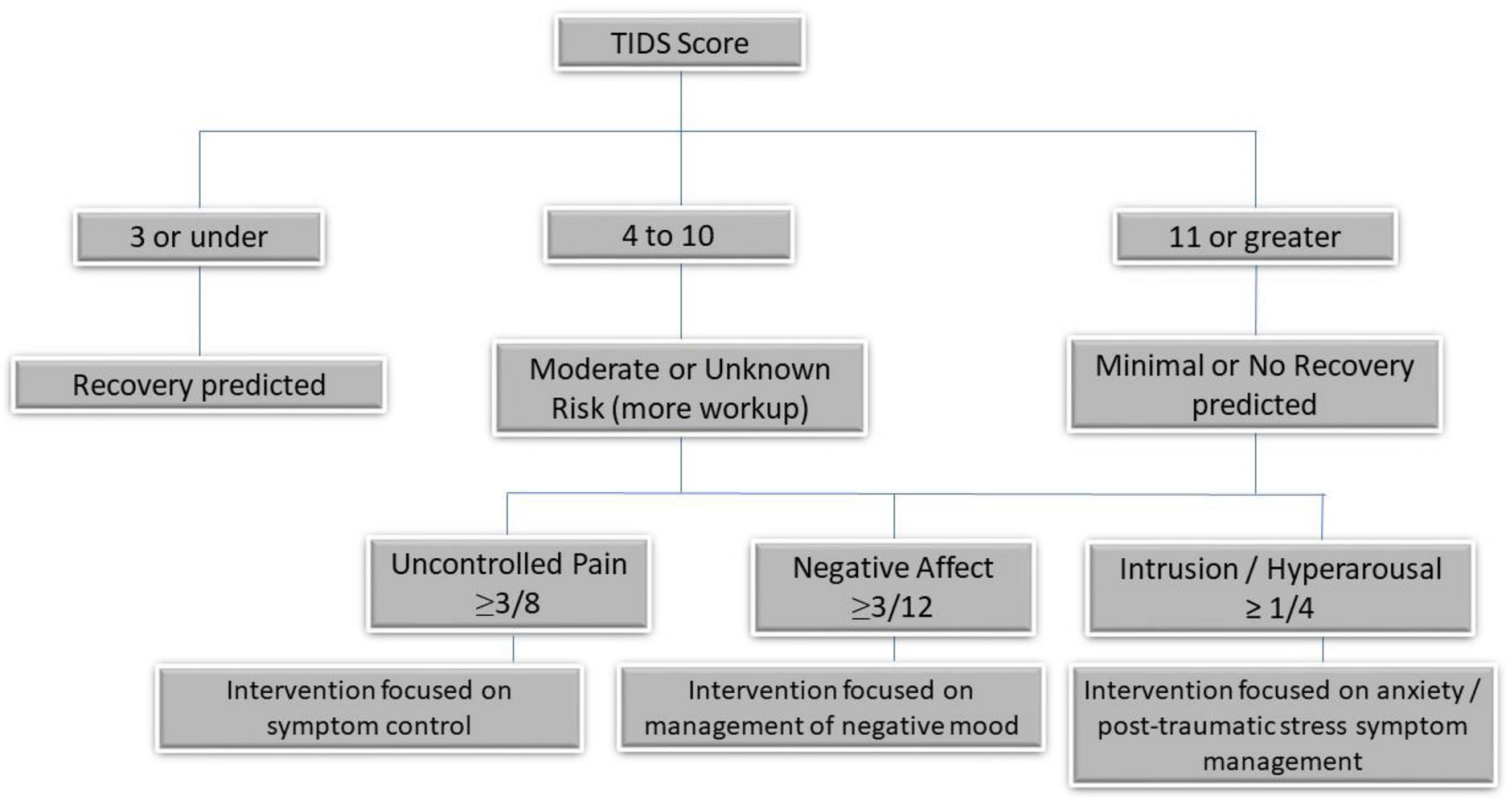
Clinical decision-making aid based on the theoretical foundations of each TIDS subscale.

To facilitate translation Table 5 presents a range of different cut scores, including positive and negative predictive values and likelihood ratios. Scores of ≤3/24 (capturing 24.1% of the sample) and ≥11/24 (capturing 27.2% of the sample) were optimal for predicting recovery or persistent problems, respectively. Using these cut scores, the TIDS accurately classified 87% (%Interference trajectory) to 98% (Pain trajectory) of those in the low risk / rapid recovery classes, and 55% (12-month point interference) to 68% (%Interference trajectory) of those in the high risk / non-recovery classes. Overall accuracy was: 76% (%Interference trajectory), 78% (Pain Severity trajectory), and 75% (12-month point %Interference score). For those deemed high risk, optimal subscale scores were: Uncontrolled pain ≥3/8 (Sn = 0.74, Sp = 0.53), Negative Affect ≥3/12 (Sn = 0.71, Sp = 0.58), Intrusion/Hyperarousal ≥1/4 (Sn = 0.71, Sp = 0.68). Fig 1 provides a decision-making aid created by these results, including broad-based management strategies constructed on the theoretical foundations of each TIDS subscale.

**Table 5:** A range of useful cut scores on the TIDS total for predicting interference recovery trajectories, Pain Severity recovery trajectories, and distal 12-month Interference scores.

Cut score	Sn	Sp	PPV	NPV	PLR	NLR
*%Interference Recovery Predicted (Rapid or Delayed)*						
**≤3**	**0.35**	**0.92**	**0.87**	**0.49**	**4.59**	**0.70**
≤4	0.43	0.87	0.83	0.51	3.25	0.66
≤5	0.52	0.81	0.80	0.54	2.78	0.59
≤6	0.59	0.75	0.77	0.55	2.32	0.55
*Minimal or No %Interference Recovery Predicted*						
≥9	0.65	0.75	0.64	0.76	2.61	0.47
≥10	0.60	0.80	0.67	0.75	2.98	0.50
**≥11**	**0.51**	**0.83**	**0.68**	**0.71**	**3.06**	**0.59**
≥12	0.47	0.86	0.70	0.71	3.49	0.61
≥13	0.40	0.90	0.73	0.69	4.05	0.67
*Rapid Pain Recovery Predicted*						
**≤3**	**0.27**	**0.97**	**0.98**	**0.21**	**9.29**	**0.75**
≤4	0.33	0.94	0.97	0.22	5.63	0.71
≤5	0.43	0.94	0.97	0.25	7.31	0.61
≤6	0.49	0.85	0.94	0.25	3.36	0.69
*Minimal or No Pain Recovery Predicted*						
≥9	0.76	0.65	0.30	0.93	2.16	0.36
≥10	0.71	0.69	0.31	0.92	2.29	0.43
**≥11**	**0.71**	**0.76**	**0.37**	**0.93**	**2.96**	**0.39**
≥12	0.65	0.79	0.38	0.92	3.09	0.45
≥13	0.50	0.83	0.36	0.89	2.87	0.61
*52-week %Interference ≤5% Predicted*						
**≤3**	**0.33**	**0.94**	**0.95**	**0.30**	**5.83**	**0.71**
≤4	0.40	0.91	0.94	0.32	4.71	0.65
≤5	0.51	0.91	0.95	0.36	5.94	0.54
≤6	0.57	0.86	0.93	0.38	3.99	0.50
*52-week %Interference ≥20% Predicted*						
≥9	0.77	0.75	0.48	0.91	3.03	0.31
≥10	0.71	0.80	0.52	0.90	3.54	0.36
**≥11**	**0.63**	**0.84**	**0.55**	**0.88**	**3.98**	**0.44**
≥12	0.57	0.88	0.59	0.87	4.65	0.49
≥13	0.46	0.90	0.59	0.84	4.74	0.60

Sn = Sensitivity, Sp = Specificity, PPV = Positive Predictive Value, NPV = Negative Predictive Value, PLR = Positive Likelihood Ration, NLR = Negative Likelihood Ratio.

## Discussion

We have presented a robust set of analyses to further explore the predictive utility of the new TIDS risk screening tool with a focus on identifying meaningful cut scores to optimize translation into practice. Consistent with the initial validation study in which baseline TIDS scores showed potentially important associations with 12-week outcomes, this analysis expanded the sample size (from n = 72 to n = 224), sampling frame (including participants from two countries), and the time delay over which outcomes were predicted (from 12 weeks to 12 months). As a single 12-item prognostic tool, the TIDS showed significant ability to predict variance in 12-month scores, and cut scores were identified that predicted the trajectory of either Pain Interference or Pain Severity with ≥76% accuracy.

While the linear relationships are interesting from an empirical perspective, the nomination of meaningful cut scores will allow clinicians to make informed decisions about the most likely recovery trajectory for individual patients. Using the thresholds of ≤3/24 for low risk and ≥11/24 for high risk, the TIDS Total score compares favorably with other risk stratification protocols. For example, predicting a good recovery in those who score ≤3/24 ranged from 87% to 98% accurate on the TIDS, compared to 71% accurate in the WAD risk stratification tool derived by Ritchie and colleagues [13], and a similar AUC as the new Musculoskeletal Health Questionnaire (MSK-HQ) [10] when discriminating between improved and not improved overall effect (MSK-HQ improved vs. not improved: AUC = 0.81, 95%CI 0.78, 0.85; TIDS Rapid vs. No Recovery: AUC = 0.85, 95%CI 0.79, 0.91). On the high-risk end, the predictive accuracy of the TIDS for those scoring ≥11/24 ranged from 55% (12-month Distal Outcome, where chance = 16.8%) to 68% (%Interference trajectory, where chance = 40.6%), compared to 71% in the WAD risk tool. It appears that the TIDS is more useful for identifying those at low risk than it is for identifying those in the higher risk category. Accuracy for the high-risk category could have been improved by choosing a higher threshold score for ‘risk’ (see Table 5) but we were cognizant of the implications of making estimation errors on this tool. If the TIDS was used as a screening tool to identify those acutely injured patients who should most appropriately be offered early access to targeted, possibly multimodal or interprofessional intervention, we were most concerned about the implications of being wrong when categorizing someone as low risk. In that error, someone who might benefit from early targeted intervention would be less likely to receive it. An error in labeling someone as higher risk was more acceptable assuming the implication would be offering rehabilitation intervention when none was needed. We acknowledge however this is not a universally correct assumption, and there could be harm to patients in being labeled as ‘at risk’ when they really are not (e.g., willingness of funders to pay for rehab or clinicians to provide it). Given the current state of rehabilitation for acute trauma, we feel justified in prioritizing accuracy in the lower risk estimation, but the additional cut scores are offered for those who are more sensitive to misclassification of the high-risk patient. We are confident the predictive value for identifying high risk patients will continue to improve as additional metrics are built into predictive models.

The TIDS Total score explained significant unique variance in outcomes 12 months later even after controlling for baseline scores on those same outcomes, and in place of the baseline score for pain severity in predicting that outcome. This is noteworthy in that prior reviews and meta-analyses have consistently found high baseline pain severity to be a consistent predictor of a poor outcome [19, 20]. One interpretation of this is that it is not the number on a 0–10 pain scale itself that is problematic, but perhaps what that number has represented. That number may have represented trauma-related distress such as is measured with the TIDS, and when entered in the same model herein it was the TIDS that explained the greater significant variance. It is also worth noting that neither age nor BMI predicted any of the outcomes in this study, a finding that appears to be in contrast to prior work [13, 20].

The TIDS total score has now shown significant ability to discriminate between 3 different recovery trajectories or outcomes classes. Prior risk screening protocols [13] and meta-analyses [20] have indicated a strong predictor of persistent functional interference at follow-up is functional interference score at baseline. Trajectories in our data could partly be predicted by acute Interference score alone, if only the Rapid Recovery class was of interest. However, the Delayed Recovery and the Minimal or No Recovery trajectories showed nearly identical interference scores at inception [15]. Relying only on baseline interference score to predict outcome would misclassify approximately ¼ of this group. In contrast, the TIDS Total score differed across all 3 trajectory classes, potentially offering clinicians the ability to identify those with high initial reports of Pain Interference *and* who are still likely to recover by 12 months. Additionally, the subscales provide some guidance for early intervention based on the most likely drivers of risk categories; clinicians can use the subscale scores to make informed decisions regarding the need for more control over pain, positive mood interventions, or post-traumatic stress management techniques. While targeted investigations are required to determine if such decisions change outcomes, these different approaches make at least theoretical sense.

The temporal component of the design and analyses lends some support to causation, though more work is needed before causal pathways can be stated with confidence. Models such as the fear-avoidance model [21] and a newer stress-diathesis model [22] provide some potential mechanistic explanations for how acute distress could lead to the genesis of persistent problems, whether through avoidance and disuse or through maladaptive stress system response. Note that the field has yet to demonstrate consistent evidence of reversibility (that reducing distress improves the outcome). To date there has been very little support of early risk-targeted intervention, though some evidence is starting to accrue [23, 24]. If the TIDS can be used to identify low risk patients, and can further provide potential risk categories by use of the subscales, we suggest that it can be useful as a screening tool to optimize assay sensitivity of intervention trials that specifically target pain control, negative affect, or post-traumatic distress.

### Critical reflexivity

In response to recent calls for more critical reflexivity in rehabilitation research [25], we offer this short reflection to address our own biases and assumptions, anticipated benefits, and unintended harms of the work. The research assumes that early identification of those at greatest risk of poor recovery will improve outcomes through targeted early intervention and prevention strategies. The exclusive use of a self-report measures (the TIDS) for this purpose is consistent with the current direction in the field, but this assumes these tools are valid and commensurate with the actual cognitions of the participants. The design of this and much of the research in the field is also predicated upon a presupposition that pain severity or functional interference are equally important outcomes for all people, though qualitative evidence indicates they are not [26–28]. Readers, and those using such risk screening protocols must recognize that, while the instructions often given to patients is ‘there are no wrong answers’, the identification and use of thresholds and cut scores to categorize patients does indeed mean there are ‘wrong’ answers, or answers that may impact care in potentially undesirable ways. The vision of such work is that causal mechanisms can be identified, intervention provided, and recovery outcomes are improved. However, a potential harm of such work is that patients get labelled as ‘high risk’ without any clear or effective strategy for intervention. We are sensitive to the effects of some models of chronic pain that place the reasons for chronicity firmly within the patient themselves (e.g. catastrophization of pain leading to chronicity), having the effect of potentially labeling those who transition to chronic pain as possible weak-willed or misinformed, rather than more outwardly focused sociopolitical drivers of health and wellness. From this perspective we have intentionally chosen to focus on ‘ruling out’ from ongoing care the low-risk patient while offering less confidence in high risk, hoping that through this simple 12-item tool those who are low-risk can be identified to reduce iatrogenic disability [29] while those not deemed low risk can undergo additional workup.

### Limitations

The most notable threat to internal validity is the most notable benefit for external validity, being the combination of two different cohorts, from two different countries, that used two different interference-based outcome measures. We have conducted 3 additional tests of equivalency (mean Interference at entry, correlations with a common metric, and prognostic functioning all when stratified by cohort) and also included ‘region of injury’ as a variable in the regression analysis to control for potential systematic differences in region and outcomes. Collectively we believe these are robust approaches to establishing acceptability for combining outcomes, though acknowledge this opinion will not be shared by all readers. A 39% rate of missing 12-month data was also a potential issue, though we have addressed that through MLE-based GMM and lines of best fit to estimate the missing values, an approach shown to be adequately valid in our prior trajectories derivation manuscript [15]. Finally, consistent with any study that relies on participant self-report data, there is no way to determine if respondents gave adequate attention to the tools. Fortunately, the large amount of data collected allowed triangulation of recovery status across metrics (e.g. work status, ongoing health services, pain, disability, and negative affect), none of which suggested wildly disparate responses across tools (not shown).

## Conclusion

We have conducted a robust prognostic evaluation of the new TIDS tool, intended as a patient-reported screening tool to identify high or low risk of poor recovery after non-catastrophic MSK injury in adults within 4 weeks of symptom onset. The tool appears to function well in this expanded and mixed cohort compared to the initial validation study and appears to function equally well across patient-level variables such as sex, age, and BMI. Suggested cut scores (≤3 and ≥11) appear to hold value in predicting outcome trajectories or end points for both pain-related functional interference and pain severity, and the subscales provide opportunity for more granular exploration of the reasons for risk. A decision tree has been provided to facilitate transition to clinical practice, though the cut-scores presented are only suggestions and a full table of useable cut-scores and associated predictive value has been provided.
